# Insight into the essential role of the *Helicobacter pylori* HP1043 orphan response regulator: genome-wide identification and characterization of the DNA-binding sites

**DOI:** 10.1038/srep41063

**Published:** 2017-01-23

**Authors:** Simone Pelliciari, Eva Pinatel, Andrea Vannini, Clelia Peano, Simone Puccio, Gianluca De Bellis, Alberto Danielli, Vincenzo Scarlato, Davide Roncarati

**Affiliations:** 1 Department of Pharmacy and Biotechnology (FaBiT), University of Bologna, Italy; 2 Institute of Biomedical Technologies, National Research Council, Segrate, Milan, Italy; 3 Doctoral School of Molecular and Translational Medicine, University of Milan, Milan, Italy

## Abstract

Many bacterial regulatory genes appear to be dispensable, as they can be deleted from the genome without loss of bacterial functionalities. In *Helicobacter pylori*, the hp1043 gene, also known as *hsrA*, is one of the transcriptional regulator that is essential for cell viability. This gene could not be deleted, nor the amount of protein modulated, supporting the hypothesis that HP1043 could be involved in the regulation of crucial cellular processes. Even though detailed structural data are available for the HP1043 protein, its targets are still ill-defined. Using Chromatin Immunoprecipitation-sequencing (ChIP-seq), one of the most powerful approaches to characterize protein-DNA interactions *in vivo*, we were able to identify genome-wide several new HP1043 binding sites. Moreover, *in vitro* DNA binding assays enabled precise mapping of the HP1043 binding sites on the new targets, revealing the presence of a conserved nucleotide sequence motif. Intriguingly, a significant fraction of the newly identified binding sites overlaps promoter regions controlling the expression of genes involved in translation. Accordingly, when protein translation was blocked, a significant induction of almost all HP1043 target genes was detected. These observations prompted us to propose HP1043 as a key regulator in *H. pylori*, likely involved in sensing and in coordinating the response to environmental conditions that provoke an arrest of protein synthesis. The essential role of HP1043 in coordinating central cellular processes is discussed.

*Helicobacter pylori* is a major pathogen, highly widespread among the human population, and it has been recognized as class I carcinogen by World Health Organization. It is considered the primary cause of severe gastrointestinal diseases such as peptic ulcer, gastric adenocarcinoma and MALT lymphoma^1^,^2^. The ability of this Gram-negative bacterium to colonize the harsh stomach niche and to establish a persistent infection depends on the coordinated expression of housekeeping genes as well as virulence factors that allow the pathogen to adapt to environmental conditions and to counteract host-defence mechanisms. A peculiar feature of the small-sized (1.66 Mb) *H. pylori* genome is the relative scarcity of genes encoding regulators of transcription. To date, only 17 transcriptional regulators have been identified and characterized to different extents^3^. Besides 3 σ factors (the housekeeping σ^80^ and the alternative RNA polymerase sigma subunits σ^54^/σ^28^, both involved in the transcription of flagellar genes) and 4 transcriptional repressors involved in metal homeostasis (Fur and NikR) or stress response (HrcA and HspR), *H. pylori* employs several two-component systems. Two-component systems are composed of a histidine-kinase and a response regulator. Upon signal perception, the sensor kinase catalyses its auto-phosphorylation and then transfers the phosphoryl group to a partner response regulator, a specific DNA binding protein that modulates transcription of target genes. Two-component systems are employed by *H. pylori* to coordinate gene expression in crucial cellular processes like chemotaxis (CheA/CheY)^4^, copper resistance (CrdS/CrdR)^5^, flagellar regulation (FlgS/FlgR)^6^, and acid acclimation (ArsS/ArsR)^7^. Intriguingly, *H. pylori* genome harbours 2 genes, named *hp1043 (HPG27_RS02035*) and *hp1021 (HPG27_RS02145*), encoding for two so-called “orphan response regulators”, as their partner sensor kinases are missing. HP1043, in particular, appears to be essential for cell viability. The *hp1043* gene, in fact, could not be deleted unless a second gene copy was integrated into the *H. pylori* chromosome^8^. The HP1043 regulator belongs to the OmpR family with a highly degenerate receiver sequence incapable of being phosphorylated. Different biochemical and structural studies suggest that HP1043 could exert its function in a phosphorylation-independent manner and it could be classified as belonging to a new response regulator family^8^,^9^. NMR-spectroscopy and X-ray crystallography suggested that HP1043 exists as a symmetric dimer, with two functional domains, an N-terminal regulatory domain and C-terminal DNA-binding domain. The dimer appears to be stable in solution in the un-phosphorylated state^10^. Even though detailed structural data are available for HP1043, the target genes bound and regulated by this regulator are still ill-defined. Specifically, to date only three HP1043 genomic binding sites have been characterized at the molecular level. *In vitro* DNA binding studies demonstrated that HP1043 binds its own promoter and the promoter region of *tlpB*, a gene encoding a methyl-accepting chemotaxis protein^11^. Moreover, in a recent study we demonstrated binding of HP1043 to the promoter region of *cncR1*, a small regulatory RNA of *H. pylori* G27 involved in the opposite modulation of motility and adhesion to host cells^12^. The impossibility of generating a knock-out mutant for *hp1043* gene, or even of modulating the amount of HP1043 protein in the cell, has hampered the detailed characterization of its regulatory function^11^. Initially proposed as a regulator of cell cycle-related functions^11^, two recent studies attempted a link of HP1043 to homeostatic stress control (and named it HsrA for homeostatic stress regulator) of the bacterial cell and to a role in oxidative stress defence and nitrogen metabolism^13^,^14^. While gel mobility shift experiments support direct binding of HP1043 (HsrA) to the *porGDAB* promoter region, the regulation of other genes by HP1043 was inferred solely by the finding of a putative binding sequence^13^,^15^.

In the present study we report the identification of HP1043 (HsrA) DNA targets *in vivo* and the characterization of selected binding sites *in vitro*. The combination of recent advances in deep sequencing coupled with well-established Chromatin Immunoprecipitation (ChIP) protocols for bacterial transcription factors, establishes ChIP-seq as one of the most powerful approaches to identify genome-wide all the *in vivo* binding sites of a regulator of interest. The ChIP-seq analysis here presented allowed us to identify several new *in vivo* HP1043 binding sites. Moreover, *in vitro* protein-DNA binding assays enabled precise mapping of the HP1043 binding sites on new targets, whose analysis revealed the presence of a conserved nucleotide sequence motif. Interestingly, a significant fraction of the newly identified motifs overlaps promoters associated to genes involved in the process of translation. Accordingly, a stress signal leading to the arrest of protein synthesis, resulted in a significant induction of almost all HP1043 target genes. These observations prompted us to propose HP1043 (HsrA) as a key regulator in *H. pylori*, likely involved in sensing and in coordinating the response to environmental conditions that provoke an arrest of protein synthesis.

## Results

### Genome-wide binding of HP1043 (HsrA)

**Figure 1:**
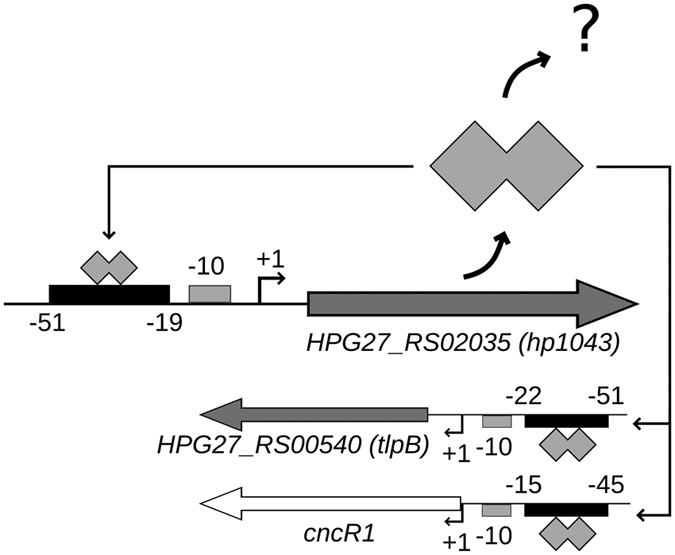
Schematic representation of the *hp1043* locus in *H. pylori* G27 along with the previously reported HP1043 binding sites. The binding sites of HP1043 are represented with black boxes, positions are relative to the transcription start sites. The transcriptional regulator, represented by a polyhedral shape, binds the promoter region of three target genes: *hp1043* and *tlpB*^11^, and *cncR1*^12^. Protein coding genes are depicted with grey block arrows, the small non-coding *cncR1* RNA gene with a white block arrow. Transcription start sites are indicated with a bent arrow marked +1, the −10 region with grey boxes.

**Figure 2:**
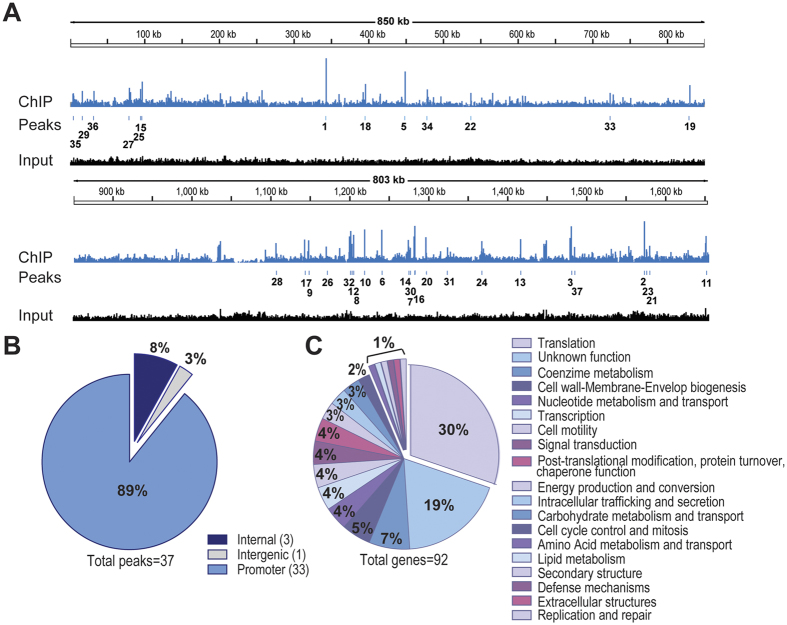
Genome-wide binding analysis of HP1043. (Panel A): genome-wide binding of HP1043 determined by ChIP-seq. The base count data, deriving from sequencing reads alignment on *H. pylori* G27 genome, of immunoprecipitated sample (labelled “ChIP”, light blue track) and control sample (labelled “Input”, black track) are represented; the position of the HP1043 binding sites, identified by peak-calling analysis, are highlighted by vertical bars and labelled according to the peak number. (Panel B): pie chart summarizing the positional analysis of the HP1043 binding sites relative to annotated genes. (Panel C): analysis of the functional categories of genes associated with novel HP1043 binding sites, identified by ChIP-seq.

To date, the exact positions of only three HP1043 genomic binding targets, located on the promoter regions of *hp1043, tlpB* and *cncR1* genes, have been characterized in details and they are represented in Fig. 1. To identify genome-wide all the HP1043 targets and thus obtain an unbiased overview of HP1043 regulatory role in *H. pylori,* we applied a ChIP-seq approach *in vivo*. In particular, liquid-grown *H. pylori* G27 wild type cells were cross-linked, sonicated and HP1043 protein-DNA complexes were immunoprecipitated (IP) with a specific polyclonal α-HP1043 antibody; at the same time a control sample (Input), deriving from sonicated but non-immunoprecipitated DNA, was prepared and used to estimate the background. A minimum of 2 million reads, with optimal mapping performances (>98.5%), for each sample and biological replicate were obtained (Supplementary Table S1) and used to generate the genome-wide binding profiles visualized in Fig. 2A. Combining Homer2 peak calling and Irreproducible Discovery Rate (IDR) procedure, we identified a set of 37 highly reproducible peaks (Fig. 2A and Table 1). These putative binding sites were the most enriched in the IP sample and highly reproducible among the two biological replicates (see Methods for a details). Afterwards, the peaks identified were annotated according to the *H. pylori* G27 RefSeq annotation (GCF_000021165.1), and cross-mapped to the transcription start sites and ncRNAs defined in strain 26695^16^ (see Methods). Specifically, binding sites centred between position −150 and +50 with respect to a transcription start site were considered associated to a promoter region, while those centred outside of this positional range were classified as internal or intergenic on the basis of the peak central position (Table 1). According to this classification, 89% of the identified HP1043 binding sites (33/37) were associated to promoters, hence in a canonical position to exert a regulatory function, and about 1/3 of them were bidirectional promoters. Three out of 37 (8%) of the peaks mapped in internal regions, while only one single peak (3%) was classified as intergenic (Fig. 2B). Interestingly, several strongly enriched peaks were located in proximity of tRNAs genes (bolded in Table 1, Supplementary Table S2).

Among HP1043 target genes, we found the small RNA *cncR1*, previously demonstrated to be bound by this regulator^12^, while the other three known targets (i.e. *hp1043* itself, *tlpB*^11^ and *porG*^14^), were not present in the top scoring list. However, two regions mapping nearby the promoters of *hp1043* and *porG* were identified as enriched by the peak caller and are present in the full list of 107 peaks identified by Homer2 (Supplementary Table S3). These peaks were not included in the top list only because of the stringent criteria adopted to evaluate the peak calling reproducibility. This observation suggests that we may find some authentic and reliable HP1043 binding sites also among some of the less reproducible ChIP-seq peaks. For example, inspection of the latter peaks allowed to pinpoint an enriched region, present in both replicas, positioned on the *tlpB* promoter. Then, to obtain a general view of HP1043 regulatory function, each promotorial binding site was associated to the downstream operon. A functional enrichment analysis was then performed to determine if the genes included in the operons belonged to specific functional categories. For this purpose, protein coding genes were classified using Clusters of Orthologous Groups of proteins (COGs) database^17^, while tRNAs and rRNAs were included to the translation category. According to this analysis, it turned out that a significant fraction (30%) of genes, associated to a HP1043 promotorial binding site and likely controlled by the regulator, is involved in the process of protein translation (Fig. 2C). Specifically, the promoters of several tRNA and rRNA genes, as well as of genes encoding ribosomal proteins (HPG27_RS05725/*rps16*, HPG27_RS06525/*rpl36*, HPG27_RS05215/*rps1*, HPG27_RS05705/*rpl19*, HPG27_RS07170/*rpl34*) or involved in rRNA and ribosome maturation and assembly (HPG27_RS05770/*ybeY* endoribonuclease and HPG27_RS06295/*frr* ribosome recycling factor) were targeted *in vivo* by HP1043. Strikingly, in the same analysis we identified HP1043 binding sites associated to genes involved in other fundamental cellular processes like energy production and conversion (flavodoxin, HPG27_RS05775/*fldA*; ATP synthase C chain, HPG27_RS06075/*atpE*; cytochrome c553, HPG27_RS06145/*cytc553*), as well as RNA transcription (HPG27_RS00460/*rpoD*, coding for the RNA polymerase housekeeping σ^80^ factor^18^). In this respect, we noticed that one more RNA polymerase gene, HPG27_RS06505/*rpoA*, encoding the RNA polymerase α subunit, is included in an operon likely controlled by HP1043. This last observation was manually curated because in the last version of *H. pylori* G27 genome annotation (GCF_000021165.1) this gene is mis-annotated to pseudogene. Overall, from the above data we can infer that HP1043 may represents a key regulator in *H. pylori*, involved in the control of crucial cellular processes, such as transcription and translation.

### Validation of novel HP1043 targets

**Figure 3:**
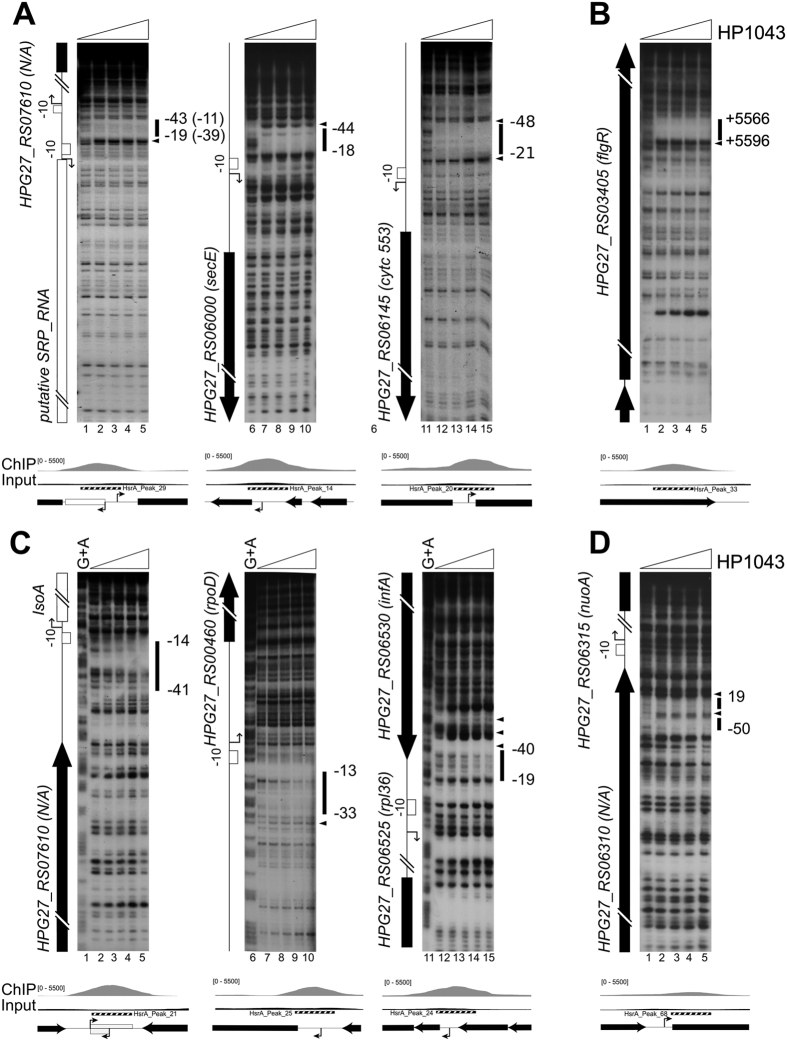
*In vitro* binding of purified recombinant HP1043 protein on selected genomic regions identified by ChIP-seq. In panel A high affinity promoter binding sites are shown, in panel B footprinting validation of an intragenic binding site is reported, in panel C low affinity promoter binding sites are represented and in panel D footprint validation of a binding site not included in the top scoring peak list. Radiolabelled DNA probes, harbouring the genomic regions of interest, were incubated with increasing concentrations of purified HP1043 (0, 1.7, 3.3, 6.6, 13.3 μM of dimeric HP1043 from left to right in each experiment) and subjected to DNase I digestion. Purified DNA fragments were separated on a polyacrylamide denaturing gel along with a G + A sequence reaction ladder (Panel C and data not shown) to map the binding sites. On the left of each autoradiograph, a schematic representation of the genomic region is drawn, with block arrows depicting coding sequences (black arrows or blocks) or putative sRNA (white arrow). Bent arrows represent the transcriptional start sites identified in *H. pylori* strain G27 by primer extension analyses (Supplementary Fig. 3S), white boxes the −10 promoter sequence. Notably, the initiation of RNA transcription at the analysed promoters is conserved between strain G27 and 26695. Black vertical lines on the right of each autoradiograph represent regions of protection from DNase I digestion, while black arrowheads highlight DNase I hyper-sensitive sites. Numbers refer to the positions with respect to the transcription start site (or with respect to the ATG translational start codon for the internal binding site on HPG27_RS03405). For the binding site mapped in the intergenic region between HPG27_RS00110 and putative SRP_RNA genes, HP1043 binding positions are reported with respect to the transcriptional start site of both genes (with no brackets for putative SRP_RNA, with brackets for HPG27_RS00110). Below each autoradiograph, a magnification of base-count data, deriving from ChIP-seq (as in Fig. 2A) of the genomic regions of interest, is reported together with a schematic representation of genes and features of the locus.

To confirm HP1043 binding sites identified by ChIP-seq, a subset of them was selected and used as probes for *in vitro* binding to a purified recombinant HP1043 protein in DNase I footprinting experiments. Among the 37 putative highly confident HP1043 targets (Table 1) we selected 7 representative genomic regions: 6 binding sites mapping within promoters and 1 site falling inside a coding sequence. We also included a DNA probe covering the promoter of the HPG27_RS06315/*nuoA* gene. This putative HP1043 binding site was present in the total peak list and close to the ranking position of *hp1043*, but excluded from the top list. Similarly to some other peaks not included in the top list of Table 1, this binding site exhibits a broad region of enrichment encompassing the promoter, even if the peak centre appears mis-positioned with respect to the core promoter region. Thus, to assess if the peaks having this profile represent *bona-fide* HP1043 binding sites with a lower binding affinity rather than artifacts of the ChIP-seq experiment, we decided to validate further by DNase I footprinting one of them (Fig. 3). Consistent with ChIP-seq findings, all the 8 genomic regions selected were directly targeted *in vitro* by the purified recombinant HP1043 protein (all probes showed a clear area of DNase I protection), apparently with different relative affinities (some probes showed protection in the presence of higher amounts of HP1043 protein). Probes bound *in vitro* with higher relative affinity are reported in Fig. 3, panel A and B, while probes bound with lower relative affinity are shown in panel C and D. Increasing concentrations of HP1043 determined the appearance of a region of protection (black box) flanked at least on one side by a single DNase I hyper-sensitive band (black arrowhead). HP1043 binding to these 4 targets protected a DNA region of about 30 bp and the protection was saturated at 1.7 μM protein concentration (Fig. 3, panel A, lanes 2, 7, 12 and panel B, lane 2); these were considered high affinity binding sites. HP1043 binding to the promoters of *isoA, rpoD, rpl36* and of *nuoA* genes (Fig. 3, panel C and D) appears at higher protein concentrations and with a slightly different binding pattern. Here the protection was saturated at a protein concentration of 13.3 μM, and again protected a region of about 30 bp flanked, only in some cases, by one or more bands of hyper-sensitivity to DNase I digestion. These were considered low affinity binding sites. Overall, the above *in vitro* footprinting assays confirm *in vivo* binding data. Notably, HP1043 binds *in vitro* exactly to the same positions of the enriched regions derived from ChIP-seq data analysis for all the top scoring peaks (Fig. 3A, B, C lower part of each panel). Moreover, the footprinting experiment reported in Fig. 3B (lanes 1 to 5) validates HP1043 intra-cistronic binding within the *flgR* coding sequence, further supporting ChIP-seq analysis and suggesting the existence of a minority of non-canonical binding sites, apparently not associated with regulatory functions.

In summary, we confirmed HP1043 binding to 8 new sites identified in *in vivo* ChIP-seq experiments and defined *in vitro* the nucleotide sequences bound by the protein.

### Molecular characterization of HP1043 (HsrA) binding to DNA

**Figure 4:**
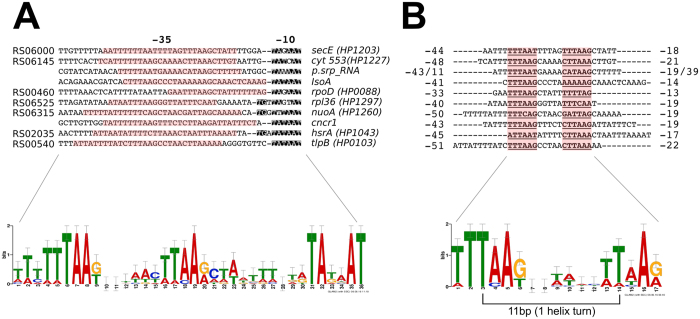
Characterization of HP1043 bound promoters (A) and definition of HP1043 consensus binding motif (B). Nucleotide sequence motifs were identified by using GLAM2 software^19^, forcing strand specific alignment and maintaining all the default parameters to obtain the weblogos. Panel A, nucleotide sequence alignment (upper part) and weblogo (lower part) relative to 10 promoter regions bound by HP1043. Specifically, DNA sequences spanning from the transcriptional start site (+1) to position −70 of the 7 genes analysed by footprinting (Fig. 3A, C, D) were aligned together with the same DNA regions upstream *tlpB, cncR1* and *hp1043* genes. Nucleotides matching the consensus −10 box and the extended TG motif are highlighted in black, while DNA regions protected by HP1043 binding, as defined by DNase I footprintings (Fig. 3), are highlighted in red. Names reported on the left of each sequence refer to the new *H. pylori* G27 genome annotation, while names on the right of each sequence report the common gene name and, in brackets, the name referred to the *H. pylori* 26695 genome annotation. Panel B, nucleotide sequence alignment of the 10 protected regions in DNase I footprintings (Fig. 3), mapping in the promoters of the same genes reported in panel A. Aligned sequences and the resulting sequence logo are shown in the upper and lower part of panel B, respectively. The sequences of the two direct repeats are represented in bold and underlined; numbers flanking each sequence refer to the coordinates of the DNase footprinting protected regions (shown also in Fig. 3). In both sequence logos (lower part of both A and B panels) the height of each letter represents the relative conservation of each base.

**Figure 5:**
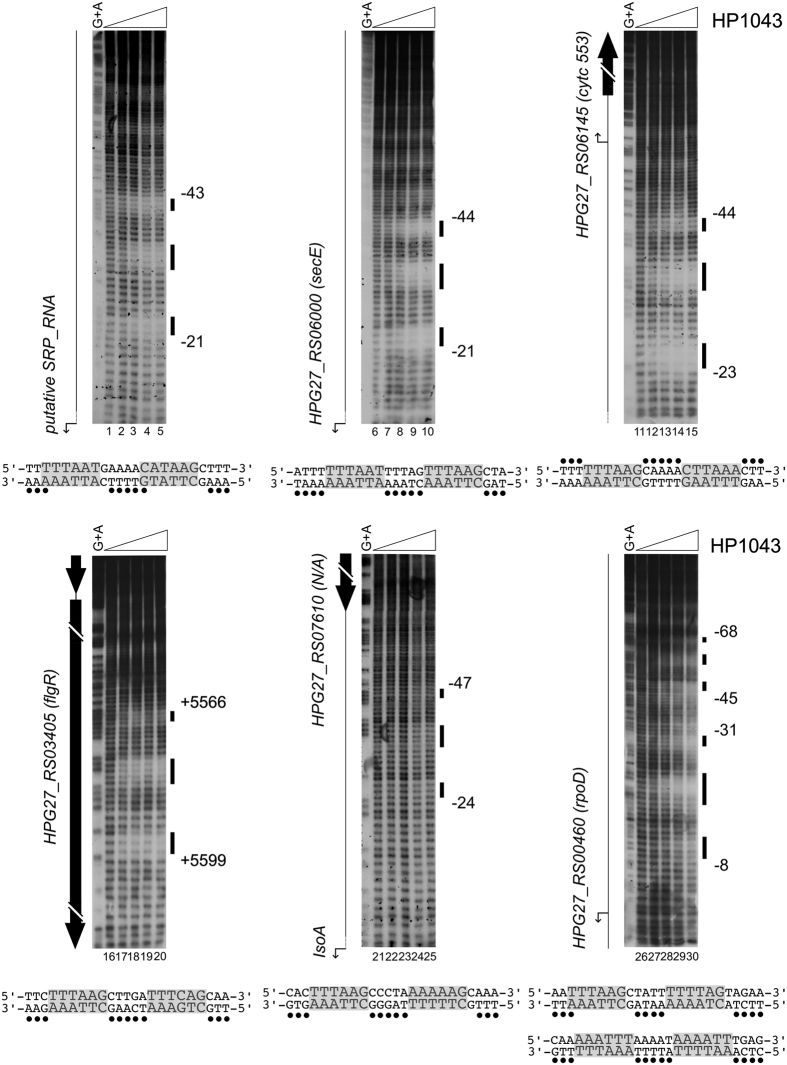
Hydroxyl-radical footprinting analysis of HP1043 binding to selected genomic regions. Radiolabelled DNA probes, harbouring the genomic regions of interest, were incubated with increasing concentrations of purified HP1043 dimer (0, 0.8, 1.7, 3.3 μM of dimeric HP1043 from left to right in each experiment) and subjected to hydroxyl-radical digestion. Purified DNA fragments were separated on a polyacrylamide denaturing gel along with a G + A sequence reaction ladder to map the binding sites. On the bottom of each autoradiograph, the nucleotide sequence of the HP1043 binding sites mapped is reported: grey-highlighted nucleotides depict the hexameric direct repeats of the binding motif, while the black dots indicate the nucleotides protected in hydroxyl-radical footprintings on the labelled DNA strands. Symbols are detailed in the legend to Fig. 3.

The DNase I footprinting data indicated that the newly identified HP1043 binding sites overlap the core promoter region and map, in all cases, immediately upstream of the −10 box (Fig. 3, panels A, C and D). This observation parallels with the positions of HP1043 binding sites on the three previously characterized target sites (Fig. 1 11,12). To get a detailed picture of the promoter sequences encompassing HP1043 binding sites, the core promoter sequences of the newly and previously validated HP1043 targets were analysed using the GLAM2 software^19^. Results are shown in Fig. 4A. The HP1043 target promoters appear to be characterized by a −10 hexamer typical of the housekeeping σ^80^-dependent transcription, preceded, in some cases, by an extended TG motif (Fig. 4A, upper part). Moreover, a sequence resembling the −35 box is lacking in these promoters, apparently replaced by two conserved AT-rich motifs separated by a spacer region (Fig. 4A, lower part). Intriguingly, the regions protected by HP1043 binding in DNase I footprinting assays (highlighted in Fig. 4A, upper part) encompass, in almost all cases (except in *rpoD*), these two conserved AT-rich motifs. To determine a possible consensus motif recognized by the HP1043 protein, we performed the same analysis using only the regions protected by HP1043 in the *in vitro* footprint experiments as input sequences. In this way GLAM2 outlined a highly conserved motif, reported in Fig. 4B. Specifically, the motif appears to be constituted by two direct repeats (TTTAAG) separated by a 5 bp-spacer, in which the first highly conserved hexamer is followed by a second less conserved one. Notably, the spacer length is conserved, positioning the centre of the two direct repeats at 11 bp distance that corresponds to 1 helical turn, suggesting that a dimer of HP1043 recognizes the DNA on the same face of the double helix. The conserved sequence motif (underlined and in bold in Fig. 4B, upper part) is either centred within the experimentally mapped binding sites (*secE, cyt553, cncR1* and *nuoA*), or shifted towards one side of the protected region (*isoA, rpoD, hp1043* and *tlpB*). Possibly, this is due to the intrinsic low level of resolution in mapping protein binding sites with DNase I footprinting assays. In fact, some regions of the DNA probes used for these assays naturally lack bands of digestion even in the sample without protein and this introduces a degree of uncertainty in defining the precise boundaries of the binding regions. Hence, to precisely map HP1043 (HsrA) binding sites and to further characterize the connection between the identified consensus motif and the intimate contacts of HP1043 with target DNA, hydroxyl-radical footprinting assays were carried out on selected targets, previously probed by DNase I footprintings (Fig. 3). Results are reported in Fig. 5. On all the promoter probes tested, HP1043 binding resulted in a periodic pattern of three short protected tracts of 3/4 nucleotides in length, separated by two non-protected regions of 5/6 nucleotides. Intriguingly, for all binding sites the central protection centers exactly within the spacer that separates the conserved direct repeats, while the other two protected DNA tracts fall immediately upstream and downstream of the direct repeats of the consensus binding motif (Fig. 5). It is worth mentioning that regions protected in hydroxyl-radical footprinting experiments reflect limited accessibility of radical ions to the DNA minor groove and, for this reason, these protected regions do not necessarily represent the portions of the probe directly contacted by the protein. Considering that HP1043 footprint regions surround the conserved direct repeats (Fig. 5), our data suggest that HP1043 could interact with the TTTAAG repeats in the DNA major groove narrowing the adjacent minor grooves which results in the protection observed *in vitro* by hydroxyl-radical footprinting. Interestingly, hydroxyl-radical footprinting analysis allowed the identification of a peculiar organization on *rpoD* promoter, which harbours multiple HP1043 binding sites. In fact, on this promoter, hydroxyl-radical footprinting assay (Fig. 5, lanes 26–30), besides the binding site already mapped with DNase I footprinting (Fig. 3C, lanes 6–10), revealed the existence of a distal HP1043 binding site, spanning positions −45 to −68 with respect to the initiation of RNA transcription. Moreover, the analysis of the DNA sequence of this distal binding site, revealed an HP1043 binding sequence highly similar to the consensus binding motif described above. The *rpoD* gene represents the first example of an HP1043 target with multiple binding sites, suggesting a complex mechanism of transcriptional regulation. In conclusion, hydroxyl-radical probing allowed to further refine the positions of HP1043 binding, providing the demonstration that the direct repeats of the proposed consensus motif are recognized by HP1043, likely through a major groove read-out mechanism.

### Genes directly targeted by HP1043 are up-regulated upon translational arrest

**Figure 6:**
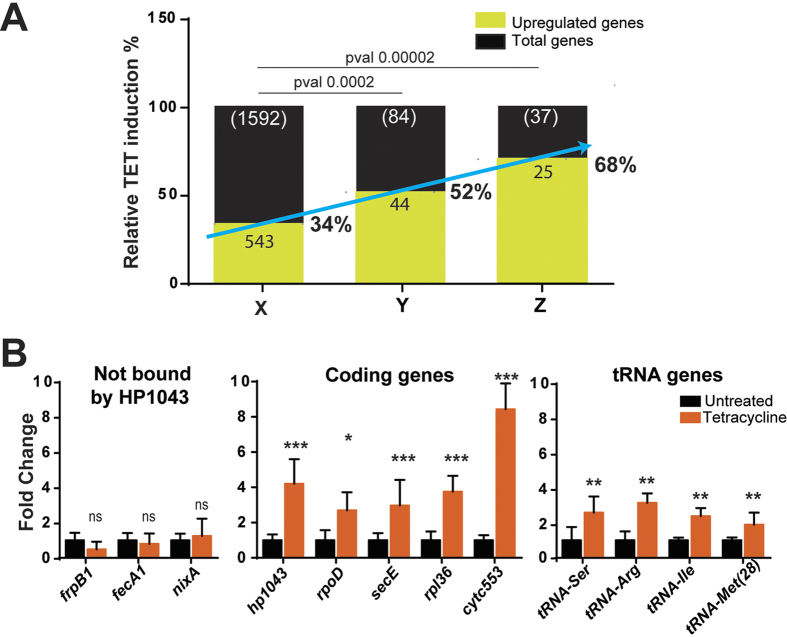
RNA-seq (A) and Real Time PCR (B) analyses of transcript level variations of novel HP1043 targets in response to translation arrest. Panel A): relative percentage of the genes upregulated after tetracycline treatment considering all annotated *H. pylori* G27 genes (column X, with exclusion of rRNA genes, depleted during sample preparation), the genes in operons under direct control of HP1043 (column Y with exclusion of 2 rRNA genes and of the genes absent in *H. pylori* annotation: 4 antisense RNA, and 2 small RNA), and the genes leading the operons under direct control of HP1043 (column Z with the exclusion of the genes indicated for column Y), respectively. The reported p-value was calculated using Fisher test. Panel B): effect of tetracycline treatment on transcript amounts of a selection of genes associated with an HP1043 binding site assessed by real time (qRT-PCR) analysis. Total RNA was extracted from cells treated or not treated with 0.5 μg/ml tetracycline for 60 min and reverse transcribed to cDNA. Transcript levels of a selection of genes not associated (panel B, left graph) or associated (panel B, central graph) with an HP1043 binding site were quantified by qRT-PCR, using the housekeeping 16 S rRNA gene as control. The same analysis was carried out on some tRNA genes (panel B, right graph) associated with HP1043 binding. Statistically significant differences were assessed by Student’s t-test (Error bars indicate the standard deviation deriving from three independent biological samples, each analysed in duplicate technical replicates). Symbols: *p value < 0.05; **p value < 0.01; ***p value < 0.001; ns, p value > 0.05, not significant.

ChIP-seq data indicated that HP1043 binds *in vivo* regulatory regions of several genes involved in translation (Fig. 2C). Hence, we hypothesized that the regulative function of HP1043 could be boosted by a signal affecting protein synthesis. To verify this hypothesis, in a preliminary survey, *H. pylori* G27 wild type cells were liquid-grown to mid-exponential phase, and HP1043 mRNA abundance was monitored at different time points after blocking protein synthesis by the addition of a sub-lethal concentration of tetracycline, which prevents aminoacyl tRNA attachment to ribosomal acceptor site^20^. Quantitative Real-Time PCR (qRT-PCR) assay revealed that the *hp1043* transcript amounts significantly increase upon tetracycline treatment, reaching a maximum induction upon 60 min of treatment (data not shown). Similar results were obtained upon treatment with chloramphenicol, which blocks ribosomes on mRNA during translation^21^. These preliminary results prompted us to further investigate the impact of translational stress on the HP1043 (HsrA) regulon. Total RNA collected from *H. pylori* wild-type cells exposed for 60 min to tetracycline and from a control culture were used to generate strand-specific cDNA libraries for RNA-sequencing analysis. Overall, differential gene expression outlined 1065 out of the 1592 total number of *H. pylori* genes to be deregulated upon antibiotic treatment. Of these, 543 genes were upregulated, representing 34% of the *H. pylori* genes (Fig. 6A, column X). However, focusing on the panel of genes belonging to all the operons controlled *in vivo* by HP1043, we observed that 52% of them were upregulated after tetracycline treatment (Fig. 6A, column Y), with a much more significant enrichment (68%) of upregulated HP1043 direct targets among the genes leading each operon (Fig. 6A, column Z). Several cellular pathways were affected by this treatment, supporting the notion that block of translation is a major challenge for bacterial cells, which respond with a wide transcriptional reprogramming of most key cellular processes. It is worth-noting that genes coding for rRNA and non-coding RNA were not included in the analysis because the first were depleted by the RNA sample preparation and the second are not annotated in strain G27.

To validate RNA-seq data, the differential mRNA levels of a subset of HP1043 targets were assayed upon tetracycline exposure by qRT-PCR analysis. Three negative controls (*frpB1, fecA1* and *nixA* i.e. three genes not targeted by HP1043) were included in the analysis and remained essentially unchanged upon antibiotic challenge (Fig. 6B, left histogram). Results reported in Fig. 6B (central histogram) show that transcript levels of selected HP1043 target genes (*hp1043, rpoD, secE, rpl36, cytc553*) increased upon tetracycline treatment, with fold variations ranging from 3- to 7-fold. Moreover, as shown in Fig. 6B (right histogram), a similar increasing trend upon translational arrest was observed for a selection of tRNA genes. These data suggest a possible involvement of the orphan response regulator HP1043 (HsrA) in the transcriptional response of *H. pylori* to environmental conditions or signals that promote arrest of protein synthesis.

## Discussion

Two-component signal transduction systems typically regulate bacterial cellular functions in response to environmental conditions through a phosphorylation-dependent process. The human pathogen *H. pylori* relies on such regulatory systems to control important cellular functions such as motility, chemotaxis, acid acclimation and copper resistance^22^. The orphan response regulator HP1043, also known as HsrA, is proven to be essential for cell growth and shows no requirement for the well-known phosphorelay scheme to be functional. In the present study, we have set up chromatin immunoprecipitation with α-HP1043 antibody followed by deep sequencing (ChIP-seq). This approach led to the identification of several new HP1043 genomic targets (Fig. 2A). To our knowledge, this is the first study that provides a genome-wide analysis of the HP1043 binding *in vivo*. Specifically, 37 genomic HP1043 binding sites were identified (Table 1), the majority of which are associated to a promoter region (Fig. 2B). A predominant fraction of genes associated to HP1043 binding encodes for proteins involved in crucial cellular functions, such as protein synthesis (tRNAs and ribosomal proteins coding genes), gene transcription (RNA polymerase subunits), and energy metabolism (operon containing genes coding for the NADH-ubiquinone oxidoreductase whole complex, as well as the ATP synthase C-chain gene promoter) (Fig. 2C, Table 1). Thus, it seems that HP1043 plays a key role for the fitness of the bacterium, which is a prerequisite for a successful infection. Moreover, our findings suggest that HP1043 regulator might represent a central regulatory switch mechanism that *H. pylori* exploits to modulate its metabolism and growth behaviour. In this respect, it is interesting to note the connection between HP1043 regulation and the arrest of translation (Fig. 6), a major challenge for bacterial cells.

Recent works by Olekhnovich and colleagues^14^,^15^ suggested an involvement of HP1043 in directly regulating a list of about 70 genes encoding for proteins with disparate functions. However, it is worth mentioning that this proposed regulon was defined by searching against the *H. pylori* genome with a consensus binding motif defined by aligning two binding sites only^15^. In our study, we have defined the HP1043 regulon by identifying *in vivo* several new direct targets and noticed that many previously proposed binding sites appear not bound in our experimental conditions.

The list of 37 HP1043 genomic binding sites (Table 1) derives from a stringent peak-calling analysis, that takes into consideration the reproducible high fold enrichment of the immunoprecipitated DNA regions with respect to the input DNA in two independent biological replicates, thus representing high-confidence candidates. The identification of this list of *bona fide* HP1043 targets likely prevented from the inclusion of some false positives, but at the same time it may have determined the exclusion of several real binding sites characterized by low affinity binding levels and/or low reproducibility among replicates. In this respect, HP1043 binding on its own promoter, a known target previously characterized at the molecular level^11^, was not included in the top list of highly significant binding sites. Moreover, we have shown that a putative HP1043 target, mapping upstream of the transcription start site of the HPG27_RS06315 gene, not included in the top list, is indeed an authentic HP1043 binding site (Fig. 3D). Hence, besides the new binding sites identified, it can be hypothesized that the HP1043 regulon may include additional members, not pinpointed by our analysis. Intriguingly, 8% of the newly identified genomic binding sites are not associated to promoters, mapping within protein coding sequences (Figs 2B and 3B). This observation poses some questions about the functional role of HP1043 binding to these internal sites. Even though we cannot exclude the existence of alternative and still ill-defined mechanisms of transcriptional regulation exerted by HP1043 bound inside coding sequences, possibly some HP1043 intracistronic binding sites could be associated with still-unknown internal promoters driving the transcription of intracistronic or antisense transcripts. Alternatively, these sites are not associated to regulation of transcription. The advent of the ‘omics’ revolution allowed the observation of binding sites not associated to regulation of several regulators, like *H. pylori* Fur repressor and *E. coli* CRP activator^23^,^24^. Even though it has recently been proposed that regulators with this behaviour may have evolved from nucleoid associated proteins^25^, validation of this hypothesis needs further investigation.

The alignment of promoter regions harbouring HP1043 binding sites (Fig. 4A) revealed that genes controlled by this regulator are transcribed by a putative vegetative σ^80^-dependent promoter with a conserved −10 box. Moreover, the −35 hexamer, typical of housekeeping promoters appeared to be lacking, consistent with previous observations in the *H. pylori* 26695 strain^16^. Intriguingly, in the subset of promoters here analysed, the −35 motif is replaced by two conserved AT-rich motifs separated by a spacer region (Fig. 4A, lower panel), overlapping HP1043 binding site. The position of HP1043 binding sites, just upstream the −10 hexamer, is typical of activators of transcription^26^,^27^. For example the binding sites of *Bacillus subtilis* PhoP transcriptional activator, belonging to the large OmpR/PhoB subfamily of response regulators to which HP1043 has previously been associated^10^, are typically centred between positions −17 to −66 of the activated promoters^28^. Accordingly, we speculate that HP1043 acts as an activator of transcription, boosting the activity of weak promoters controlling crucial genes involved in key cellular functions. Binding of HP1043 upstream of the −10 box would facilitate the contacts between the regulator and RNA polymerase, thereby stimulating initiation of transcription. This hypothesis might also be partly supported by the data summarized in Fig. 6B.

To identify sequence specific determinants for HP1043 binding, 10 nucleotide sequences protected in footprinting analyses were aligned using the GLAM2 computer program. A highly conserved motif (Fig. 4B) was identified and then proved to be bound by HP1043 through hydroxyl-radical footprintings (Fig. 5). The conserved binding motif is composed of two direct repeats (one highly conserved repeat followed by a second less conserved repeat) separated by a 5-bp spacer conserved in length. The HP1043 binding motif appears to be located on the coding strand in almost all promoters analysed. The only exception is represented by *hp1043* (Fig. 4B), in which the conserved direct repeat maps on the non-coding DNA strand. In a previous study, a portion of *hp1043* promoter was used to characterize HP1043 binding to DNA through electrophoretic mobility shift assay carried out on wild-type and mutated probes^10^. In particular, deletions of the second less conserved repeat, as well as single base mutations of highly conserved A and G of the first repeat, significantly impaired protein binding to DNA, supporting the identified HP1043 consensus binding motif.

*In vitro* characterization of HP1043 binding to selected promoters (Figs 3 and 5) revealed the existence of high affinity and low affinity binding sites. However, the comparison of the conserved direct repeats of these targets did not suggest any evident sequence feature responsible for the discriminative HP1043 binding capacity. Further characterization of the HP1043 DNA binding motif is crucial to address this point.

The above mentioned conserved spacing between the direct repeats puts the centre of the two direct repeats at 11 bp distance that corresponds to 1 helical turn, suggesting that a dimer of HP1043 recognizes the DNA on the same face of the double helix. The structure of HP1043 determined by NMR and X-ray crystallography^10^ supports this hypothesis, revealing a symmetrical dimer with two functional domains: the regulatory (dimerization) domain and the DNA-binding (transactivation) domain. In particular, the DNA-binding domains in the dimer appeared to be spaced by a distance compatible with one helix turn, allowing the interaction of each DNA-binding domain with one repeat of the conserved motif. Considering that the HP1043 regulatory domains form a symmetric dimer and that they are connected to their respective DNA-binding domains through a short 2-residues linker, it is conceivable that HP1043 forms a symmetric dimer in a head-to-head orientation with DNA. Consequently, HP1043 should be expected to contact a binding site made by an inverted repeat. Surprisingly, our sequence conservation analysis led to the identification of a binding motif characterized by two direct repeats (Fig. 4B). This observation can partially be explained considering the different conservation of the two hemi-sites. Specifically, the HP1043 binding motif appears to be constituted by a first highly conserved hexamer followed by a second less conserved repeat. The different degree of conservation between the two hemi-sites could account for a different specificity of DNA recognition of the two HP1043 monomers. A DNA recognition mechanism like this has been proposed for HpNikR, an *H. pylori* transcriptional regulator of nickel homeostasis^29^. It has been shown that a dimer of symmetrical dimers of NikR, expected to contact inverted repeats, has more affinity for binding sites that deviate from a perfect inverted repeat architecture^29^. Accordingly, it has been proposed that during the interaction between NikR and DNA target sequence, the more conserved hemi-site acts as a recognition site, while the second less conserved repeat acts as a structural (stabilizing) binding site^29^. The DNA recognition mechanism of HP1043 could be similar, with the primary recognition event taking place only on the highly conserved repeat, thereby stabilized by a weaker interaction between the second repeat and the other DNA binding domain. Another possibility is that, upon DNA recognition, the HP1043 dimer undergoes a structural reorganization, allowing a prototypical interaction between a direct repeat motif and a proper oriented dimer. Further experiments will disentangle this apparent paradox in HP1043-DNA docking mechanism.

## Methods

### Bacterial strains and culture conditions

Bacterial strains used in this study are listed in Table 2. *H. pylori* G27 wild type cells were revitalized from glycerol stocks on Brucella broth agar plates containing 5% fetal calf serum (FCS) in a 9% CO_2_–91% air atmosphere at 37 °C and 95% humidity in a water-jacketed incubator (Thermo Forma Scientific). Liquid cultures were performed in Brucella broth medium supplemented with 5% FCS in glass flasks. *E. coli* cells were grown on Luria–Bertani (LB) agar plates or LB liquid broth; when required, ampicillin was added to the medium to achieve a final concentration of 100 μg/ml.

### DNA manipulations

DNA manipulations were performed as described by Sambrook *et al*.^30^. All restriction and modification enzymes were used according to the manufacturers’ instructions (New England Biolabs). Preparations of plasmid DNA were carried out with NucleoBond Xtra Midi plasmid purification kit (Macherey-Nagel).

### Overexpression and purification of recombinant HP1043 protein

Recombinant N-terminal His-tagged HP1043 protein was overexpressed in *E. coli* and affinity-purified as previously described^11^. Purified HP1043 was dialysed against two changes of 1 × 1043 Footprinting Buffer (1 × 1043 FPB: 10 mM Tris-HCl pH 7.5; 50 mM NaCl; 10 mM MgCl_2;_ 1 mM DTT; 0.01% Igepal CA-630; 10% glycerol) for DNA-binding assays or against two changes of 1X PBS (137 mM NaCl; 2.7 mM KCl; 10 mM NaH_2_PO_4_; 1.8 mM KH_2_PO_4_; pH 7.4) for animal immunization. Protein concentration was determined by Bradford colorimetric assay (Bio-Rad) and the purity of the protein preparations was analysed by SDS-PAGE.

### Chromatin Immunoprecipitation (ChIP) with α-HP1043 polyclonal antibody

Available α-HP1043 polyclonal antibody from immunized rabbits^12^ were purified by 3 sequential precipitations with 35% saturated (NH_4_)_2_SO_4_ and subsequent resuspension in water. *H. pylori* G27 wild type cultures were liquid-grown to an OD_600_ of 1.0, harvested, crosslinked, sonicated and immunoprecipitated as previously described^12^. Briefly, protein-DNA complexes were chemically crosslinked with 1% formaldehyde, then DNA was shared by extensive sonication with Bioruptor (Diagenode). HP1043 covalently linked to target DNA fragments were immunoprecipitated by incubating the crosslinked whole cell extracts with the polyclonal α–HP1043 antibody and then capturing the resulting complexes with Protein-G-conjugated sepharose beads. Cross-linking was reverted for 6 h at 65 °C, with occasional mixing. DNA was extracted once with phenol-chloroform and further extracted with chloroform. After the addition of 1% glycogen, DNA was ethanol precipitated and suspended in 50 μl double-distilled H_2_O, as previously described^12^.

### ChIP-seq library preparation and sequencing

Illumina libraries were prepared, for each biological replicate either from 5 ng of immunoprecipitated-DNA (IPs) or from 5 ng of the Input-DNA following the Illumina TruSeq ChIP-seq DNA sample preparation protocol; then each library was sequenced on a MiSeq Illumina sequencer and 51 bp single stranded reads were produced.

### ChIP-seq data analysis

Bowtie 2 (v2.2.6)^31^ was used to align raw reads, produced from ChIP sequencing experiments, to *H. pylori* G27 genome. End-to-end mapping was performed and non-deterministic option was specified to force a single assignment of multi-mapping reads to the best scoring region (if present) or a random attribution in the case of regions with identical scores. High quality reads were then selected requiring: for uniquely mapping reads MAPQ (mapping quality) greater than 30 and alignment score greater than −10 while for multi-mapping reads alignment score was set equal or greater than −10. The quality of ChIP-Seq data was evaluated following ENCODE quality metrics (https://code.google.com/archive/p/phantompeakqualtools/) and the numerical values obtained are provided in Supplementary Table S1. The cross-correlation analysis resulted in good NSC and RSC values, obtained using the code from Dr. Kundaje at Stanford University (https://code.google.com/archive/p/phantompeakqualtools/) cited by ENCODE consortium (http://genome.ucsc.edu/ENCODE/encodeTools.html). Moreover, we obtained average PBC scores. Irreproducible Discovery Rate procedure (IDR v 2.0.2) following ENCODE guidelines^32^, and using Homer (v4.7.2)^33^ as peak caller, was performed to measure sample reproducibility and to identify consistent peaks. Homer parameters were set according to the authors’ indication for IDR calculation (-P. 1 –LP. 1 –poisson. 1), -L was set to three. The “findPeaks score” column was selected as ranking column for IDR calculations, as suggested by Homer authors. The pool of two independent input preparations was used as background for the analysis, as suggested by IDR procedure. Peaks were manually annotated to the genes having transcription start site within −150/ + 50 bp from the middle of the peak (promoter peaks), to the genes containing the middle of peak (intragenic) or to the two genes surrounding the intergenic regions containing the peak (intergenic), according to the current version of *H. pylori* G27 RefSeq annotation (GCF_000021165.1). Transcription start sites were derived by blasting the 50 bp before the initiation of transcription found by Sharma *et al*.^16^ for 26695 and controlling their coherence with our RNA-seq signals. A transcriptional start site was automatically cross-mapped when fragments matched with at least 90% identity and at least with 45/51 nucleotides in length. To further define peaks annotation, for the regions where HP1043 was bound, we considered also the fragments matching with an identity of at least 80% and an overlap of 35/51 nucleotides, if showing an increase of transcription (according to our RNAseq data) in correspondence to the cross-mapped transcriptional start site. To obtain COG^34^ annotation for the putative protein coding targets the protein accession number of those genes were submitted to CDD^35^ online database and COG alphanumeric code was converted to function according to the official COG classification. Raw data are publicly available at Sequence Reads Archive under accession number BioProject PRJNA327549.

### DNase I footprinting

Genomic regions harbouring the putative HP1043 binding sites identified by ChIP-seq analysis were PCR-amplified with oligonucleotides listed in Supplementary Table S4 and cloned in pGEM-T-Easy vector (Table 2). DNA fragments obtained by digestion with the appropriate restriction enzymes were 5′-end labelled with [γ-^32^P]-ATP and T4 polynucleotide kinase and gel purified. Approximately, 15 fmol of labelled probe were used for each footprinting reaction. DNase I footprinting experiments were performed as previously described^36^. Briefly, the labelled probes were incubated with increasing amounts of purified recombinant HP1043 protein in 1 × 1043 FPB containing 300 ng of sonicated salmon sperm DNA as non-specific competitor in a final volume of 50 μl for 20 min at room temperature. The partial digestion of the labelled probe was carried out using 0.01U of DNase I (Novagen) diluted in 1 × 1043 FPB supplemented with 5 mM CaCl_2_ and 1 mM DTT for 90 s at room temperature. Reactions were stopped with 140 μl of STOP buffer (192 mM NaOAc pH 5.2; 32 mM EDTA pH 8.0; 0.14% SDS; 64 μg/μl sonicated salmon sperm DNA), phenol-chloroform extracted and ethanol precipitated. Samples were resuspended in 10 μl of Formamide Loading Buffer (FLB: 95% formamide; 10 mM EDTA; 0.02% bromophenol blue; 0.02% xylene cyanol), denatured at 100 °C for 3 min, fractionated on a 8 M urea-6% polyacrylamide sequencing gel and auto-radiographed.

### Hydroxyl-radical footprinting

Genomic regions of interest were 5′-end labelled and purified as described above for DNase I footprintings. Hydroxyl-radical footprinting assays were performed as previously described^37^. Briefly, the labelled probes were incubated with increasing amounts of HP1043 protein in 1 × 1043 FPB_0_ (10 mM Tris-HCl pH 7.5; 50 mM NaCl; 10 mM MgCl_2;_ 1 mM DTT; 0.01% Igepal CA-630) containing 300 ng of sonicated salmon sperm DNA as non-specific competitor in a final volume of 30 μl for 20 min at room temperature. Partial digestion of the labelled probe was performed by the simultaneous addition of 2 μl each of the following solutions: 2 μl of Fe:EDTA solution (125 mM Fe (NH_4_)_2_ (SO_4_)_2_; 250 mM EDTA pH 8.0), 2 μl of 0.1 M DTT and 2 μl 1% H_2_O_2_. After a 2-min incubation, cutting reaction was stopped with the addition of 25 μl of STOP solution (600 mM NaOAc pH 5.2; 100 ng/μl sonicated salmon sperm DNA; 4% glycerol), phenol-chloroform extracted and ethanol precipitated. Samples were resuspended in 6 μl of FLB, denatured at 100 °C for 3 min, fractionated on 8 M urea-8.4% polyacrylamide sequencing gel and auto-radiographed.

### RNA isolation

*H. pylori* cultures were liquid-grown until mid-exponential phase (OD_600_ = 0.8) then treated with 0.5 μg/ml of tetracycline for 60 min. Bacterial cells were harvested and total RNA was extracted with TRI-reagent (Sigma-Aldrich), according to manufacturer’s protocol.

### RNA-seq: library preparation, sequencing and analyses

Ribosomal RNAs were depleted starting from 1 μg of total RNA from each of the conditions analysed by using the RiboZero Gram negative kit (Epicentre, Illumina). Strand specific RNA-seq libraries were prepared by using the ScriptSeq^TM^ v2 RNAseq library preparation kit (Epicentre, Illumina) starting from 50 ng of previously rRNA depleted RNA from each biological replicate and for all the conditions analysed. Then each library was sequenced on a MiSeq Illumina sequencer and 76 bp reads were produced. Bowtie 2 (v2.2.6)^31^ was used to align raw reads to *H. pylori* G27 genome with the same parameters used for ChIP-seq data. High quality reads were selected requiring: for uniquely mapping reads MAPQ (mapping quality) greater than 30 and alignment score greater than −15; for multi-mapping reads alignment score was set equal or greater than −15 rRNA depletion, strand specificity and gene coverage were evaluated using BEDTools (v2.20.1*)^38^ and SAMtools (v0.1.19)^39^ to verify the library preparation and sequencing performances (see Supplementary Table S1). Strand specific reads overlapping to the genes annotated in *H. pylori* G27 RefSeq annotation (GCF_000021165.1) for at least 50% of their length were considered to produce the raw-counts of each sample. Only rRNAs were excluded as they were depleted during the library preparation procedure. The R package DESeq2 (v1.4.5)^40^ was then used to normalize the counts and to individuate differentially expressed features showing BH adjusted p-value lower than 0.01. DESeq2 uses one of the most common strategy to normalize data after sequencing, which is the median-of-ratio method. In brief for each non variable gene the ratio of the expression level between samples is calculated, then the median ratio across all expressed genes is used as the normalization scale.

Raw data are publicly available at Sequence Reads Archive under accession number BioProject PRJNA327549.

### qRT-PCR analysis

Synthesis of cDNA and qRT-PCR analysis were carried out as previously described^41^. Briefly, after removal of contaminating genomic DNA through DNase I digestion, 1 μg of DNA-free total RNA was mixed with 50 ng of random hexamers (Invitrogen), dNTPs (1 mM each), AMV-Reverse Transcriptase (Promega) and incubated for 1 h at 37 °C for cDNA synthesis. For qRT-PCR analyses, 2 μl of the diluted (1:10) cDNA samples were mixed with 5 μl of 2X Power Up SYBR Green master Mix (ThermoFisher Scientific) and oligonucleotides specific for the genes of interest (Supplementary Table S4) at 400 nM concentration in a final volume of 10 μl. qRT-PCR was performed using the following cycling protocol: 95 °C for 2 min, then 40 cycles consisting of a denaturation for 5 s at 95 °C followed by 30 s at 60 °C (annealing and extension steps). For each real time experiment, the specificity of the reaction was checked by including a melting profile at the end of the run. Data were analysed using the ∆∆Ct method, using the 16 S rRNA gene as internal reference. qRT-PCR of 16 S rRNA on samples from cells untreated or treated with tetracycline gave overlapping amplification curves, indicating that the amount of 16 S rRNA was not changed during the time-course experiment.

## Additional Information

**How to cite this article:** Pelliciari, S. *et al*. Insight into the essential role of the *Helicobacter pylori* HP1043 orphan response regulator: genome-wide identification and characterization of the DNA-binding sites. *Sci. Rep.* **7**, 41063; doi: 10.1038/srep41063 (2017).

**Publisher's note:** Springer Nature remains neutral with regard to jurisdictional claims in published maps and institutional affiliations.

## Supplementary Material

Supplementary Information

## Figures and Tables

**Table 1:** Location of putative HP1043 binding sites.

Peak_name	start:end	Score	Class	Nearest genes
HsrA_Peak_1	342452:342626	33634,2	bidirectional promoter	nadE < **tRNA-Arg > **ilvC
HsrA_Peak_2	1570125:1570299	29290,7	promoter	> tRNA-Phe
HsrA_Peak_3	1476498:1476672	27064	promoter	16SrRNA<
HsrA_Peak_4^$^	1:126	26419	promoter	> HPG27_RS07980
HsrA_Peak_5	448262:448436	24526,6	promoter	**tRNA-Pro < **
HsrA_Peak_6	1212453:1212627	22077	promoter	fldA <
HsrA_Peak_7	1276504:1276678	21217,1	promoter	atpE<
HsrA_Peak_8	1194610:1194784	20592,6	promoter	16SrRNA<
HsrA_Peak_9	1140440:1140614	20533	bidirectional promoter	galE**<tRNA-Ile**
HsrA_Peak_10	1234366:1234540	20239,5	bidirectional promoter	nupC<**tRNA-Met > **HPG27_RS05875
HsrA_Peak_11	1648608:1648782	17967,3	promoter	> flgG2,flgG
HsrA_Peak_12	1198357:1198531	17780,6	promoter	rpS16,rpsP <
HsrA_Peak_13	1411804:1411978	17187,4	bidirectional promoter	asRNA_HPG27_RS06840< > HPG27_RS06835 (pseudogene)
HsrA_Peak_14	1268342:1268516	16391,8	promoter	secE <
HsrA_Peak_15	96262:96436	16366,7	bidirectional promoter	fabD < **tRNA-Ser**
HsrA_Peak_16	1275920:1276094	15715,5	intergenic	tRNA-Leu«tRNA-Leu
HsrA_Peak_17	1135651:1135825	15701,4	bidirectional promoter	nrdB,nrdF< > tRNA-Leu
HsrA_Peak_18	395023:395197	15492,7	promoter	**tRNA-Ser < **
HsrA_Peak_19	829599:829773	14406,8	promoter	as_HPG27_RS03935<
HsrA_Peak_20	1290665:1290839	14058,4	bidirectional promoter	hemN2,hemN< > cytc553
HsrA_Peak_21	1576835:1577009	13402,5	promoter	isoA<
HsrA_Peak_22	536813:536987	13358,6	promoter	cncR1_Hpnc2630<
HsrA_Peak_23	1573020:1573194	13228,3	internal	dnaA<
HsrA_Peak_24	1362385:1362559	12858	promoter	rpl36,rpmJ<
HsrA_Peak_25	94349:94523	11431,6	promoter	rpoD<
HsrA_Peak_26	1163325:1163499	11422,2	promoter	> asRNA_HPG27_RS05530 (Hpnc3560)
HsrA_Peak_27	79140:79314	11103,6	promoter	**tRNA-Val<**
HsrA_Peak_28	1099169:1099343	10857,3	promoter	> rps1,rpsA
HsrA_Peak_29	16420:16594	10304,9	bidirectional promoter	putative_SRP_RNA < > HPG27_RS00110
HsrA_Peak_30	1270429:1270603	10176,2	promoter	> hetA
HsrA_Peak_31	1318842:1319016	9864	bidirectional promoter	bioC < > secG
HsrA_Peak_32	1196544:1196718	9212,8	promoter	rpl19,rplS<
HsrA_Peak_33	722928:723102	8986,8	internal	> flgR
HsrA_Peak_34	478010:478184	8089,2	promoter	> hofC
HsrA_Peak_35	4434:4608	7929,2	internal	**tRNA-Lys < **
HsrA_Peak_36	31591:31765	7905,6	bidirectional promoter	HPG27_RS00165< > uspA
HsrA_Peak_37	1480392:1480566	7634,1	promoter	> rpl34,rpmH

^$^Located on G27 plasmid; Bold, feature included in the peak; >/<, target gene strand.

**Table 2:** Bacterial strains and plasmids.

Bacterial strains/plasmids	Description	Source/Reference
*Strain*		
* H. pylori* G27 wild type	Clinical isolate, wild type	42
* E. coli* DH-5α	*supE44 ΔlacU169 (ϕ80 lacZΔM15) hsdR17 recA1 endA1 gyrA96 thi-1 relA1*	43
*Plasmid*		
* *pGEM-T-Easy	Cloning vector; Amp^r^.	Promega
* *pGEM0703	pGEM-T-Easy derivative, containing a 437 bp DNA fragment corresponding to the region from 722.774 to 723.211 of *H. pylori* G27 genome amplified by PCR with oligonucleotides 0703FPF/0703FPR. This region corresponds to a portion of the coding sequence of HPG27_RS03405 (HP0703 according to 26695 annotation).	This work
* *pGEMp1203	pGEM-T-Easy derivative, containing a 302 bp DNA fragment corresponding to the region from 1.268.274 to 1.268.576 of *H. pylori* G27 genome amplified by PCR with oligonucleotides 1203FPF/1203FPR. This region encompasses the putative promoter region of HPG27_RS06000 (HP1203 according to 26695 annotation).	This work
* *pGEMp1227	pGEM-T-Easy derivative, containing a 308 bp DNA fragment corresponding to the region from 1.290.538 to 1.290.846 of *H. pylori* G27 genome amplified by PCR with oligonucleotides 1227FPF/1227FPR. This region encompasses the putative promoter region of HPG27_RS06145 (HP1227 according to 26695 annotation).	This work
* *pGEMsRNA17_18	pGEM-T-Easy derivative, containing a 426 bp DNA fragment corresponding to the region from 16.304 to 16.730 of *H. pylori* G27 genome amplified by PCR with oligonucleotides sRNA17_18FPF/sRNA17_18FPR. This region encompasses the intergenic region between putative SRP_RNA and HPG27_RS00110.	This work
* *pGEMA1.4	pGEM-T-Easy derivative, containing a 199 bp DNA fragment corresponding to the region from 1.576.846 to 1.577.045 of *H. pylori* G27 genome amplified by PCR with oligonucleotides A1.4FPF/A1.4FPR. This region encompasses the putative promoter region of *isoA* toxin/antitoxin system.	This work
* *pGEMp0088	pGEM-T-Easy derivative, containing a 275 bp DNA fragment corresponding to the region from 94.285 to 94.560 of *H. pylori* G27 genome amplified by PCR with oligonucleotides 0088FPF/0088FPR. This region encompasses the putative promoter region of HPG27_RS00460 (HP0088 according to 26695 annotation).	This work
* *pGEMp1296	pGEM-T-Easy derivative, containing a 477 bp DNA fragment corresponding to the region from 1.362.205 to 1.362.682 of *H. pylori* G27 genome amplified by PCR with oligonucleotides 1296FPF/1296FPR. This region encompasses the putative promoter region of HPG27_RS06525 (HP1297 according to 26695 annotation).	This work
* *pGEMp1260	pGEM-T-Easy derivative, containing a 242 bp DNA fragment corresponding to the region from 1.321.773 to 1.322.015 of *H. pylori* G27 genome amplified by PCR with oligonucleotides 1260FPF/1260FPR. This region encompasses the putative promoter region of HPG27_RS06315 (HP1260 according to 26695 annotation).	This work
* *pGEM0703HY	pGEM-T-Easy derivative, containing a 117 bp DNA fragment corresponding to the region from 722.983 to 723.100 of *H. pylori* G27 genome amplified by PCR with oligonucleotides 0703HYF/0703HYR. This region corresponds to a portion of the coding sequence of HPG27_RS03405 (HP0703 according to 26695 annotation).	This work
* *pGEMp1203HY	pGEM-T-Easy derivative, containing a 148 bp DNA fragment corresponding to the region from 1.268.391 to 1.268.539 of *H. pylori* G27 genome amplified by PCR with oligonucleotides 1203HYF/1203HYR. This region encompasses the putative promoter region of HPG27_RS06000 (HP1203 according to 26695 annotation)	This work
* *pGEMp1227HY	pGEM-T-Easy derivative, containing a 146 bp DNA fragment corresponding to the region from 1.290.664 to 1.290.810 of *H. pylori* G27 genome amplified by PCR with oligonucleotides 1227HYF/1227HYR. This region encompasses the putative promoter region of HPG27_RS06145 (HP1227 according to 26695 annotation)	This work
* *pGEMp0088HY	pGEM-T-Easy derivative, containing a 136 bp DNA fragment corresponding to the region from 94.431 to 94.567 of *H. pylori* G27 genome amplified by PCR with oligonucleotides 0088HYF/0088HYR. This region encompasses the putative promoter region of HPG27_RS00460 (HP0088 according to 26695 annotation).	This work
* *pGEMpsRNA17_18HY	pGEM-T-Easy derivative, containing a 129 bp DNA fragment corresponding to the region from 16.516 to 16.645 of *H. pylori* G27 genome amplified by PCR with oligonucleotides sRNA17_18HYF/sRNA17_18HYR. This region encompasses the intergenic region between putative SRP_RNA and HPG27_RS00110.	This work
* *pGEMpA1.4HY	pGEM-T-Easy derivative, containing a 189 bp DNA fragment corresponding to the region from 1.576.888 to 1.577.077 of *H. pylori* G27 genome amplified by PCR with oligonucleotides A1.4HYF/A1.4HYR. This region encompasses the putative promoter region of *isoA* toxin/antitoxin system.	This work
