# Supplementary material for: Insight into the essential role of the Helicobacter pylori HP1043 orphan response regulator: genome-wide identification and characterization of the DNA-binding sites
Source: Sci Rep. 2017 Jan 23;7:41063. doi: 10.1038/srep41063 (PMC5253667; doi:10.1038/srep41063)
Supplement: Supplementary Information [file srep41063-s1.pdf]

Insight into the essential role of the *Helicobacter pylori* HP1043 orphan response regulator: genome-wide identification and characterization of the DNA-binding sites

Simone Pelliciari[1,§], Eva Pinatel[2,§], Andrea Vannini[1,4], Clelia Peano[2], Simone Puccio[2,3], Gianluca De Bellis[2], Alberto Danielli[1], Vincenzo Scarlato[1,*] and Davide Roncarati[1,*]

[1]Department of Pharmacy and Biotechnology (FaBiT), University of Bologna, Italy. [2]Institute of Biomedical Technologies, National Research Council, Segrate, Milan, Italy.

Supplementary information

Figure S1

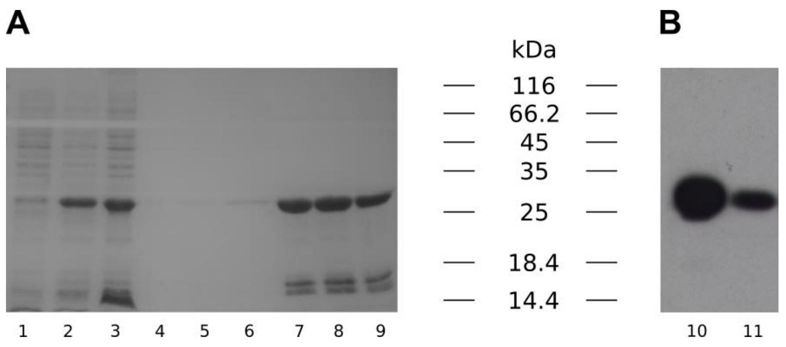

Purification steps of the recombinant HP1043 protein (panel A) and antibody specificity (panel B). A) SDS-PAGE of the recombinant HP1043 protein purification steps. Lanes as follows: non-induced total cell extract (lane 1), induced total cell extract (lane 2), soluble fraction (lane 3), 10 mM imidazole wash (lane 4), 20 mM imidazole wash (lane 5), 40 mM imidazole wash (lane 6), eluted fractions (lanes 7-9).
B) Western Blot stained with sera raised in rabbit against HP1043; lanes 10-11: 50 ng of purified protein and 10 µg of a total cell extract from *H. pylori* strain G27, respectively. A protein ladder marker (Thermo Scientific) is reported.

Figure S2

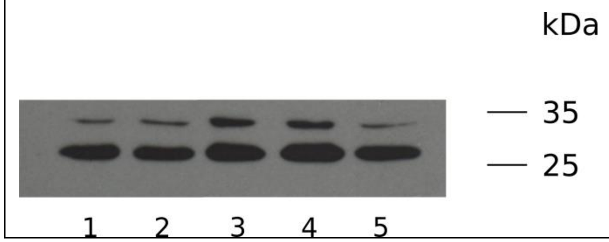

Time course experiment of *H. pylori* strain G27 treated with tetracycline. Western Blot stained with HP1043 antibody. Lane 1: 20 ng of purified recombinant HP1043 protein; lane 2: total cell extract from *H. pylori* G27 strain; lanes 3-5: total cell extract from cells treated with 0.5 µg/ml tetracycline for 30 min (lane 3), 60 min (lane 4), 90 min (lane 5). On the right side of the panel is reported a protein ladder marker (Thermo Scientific).

Figure S3

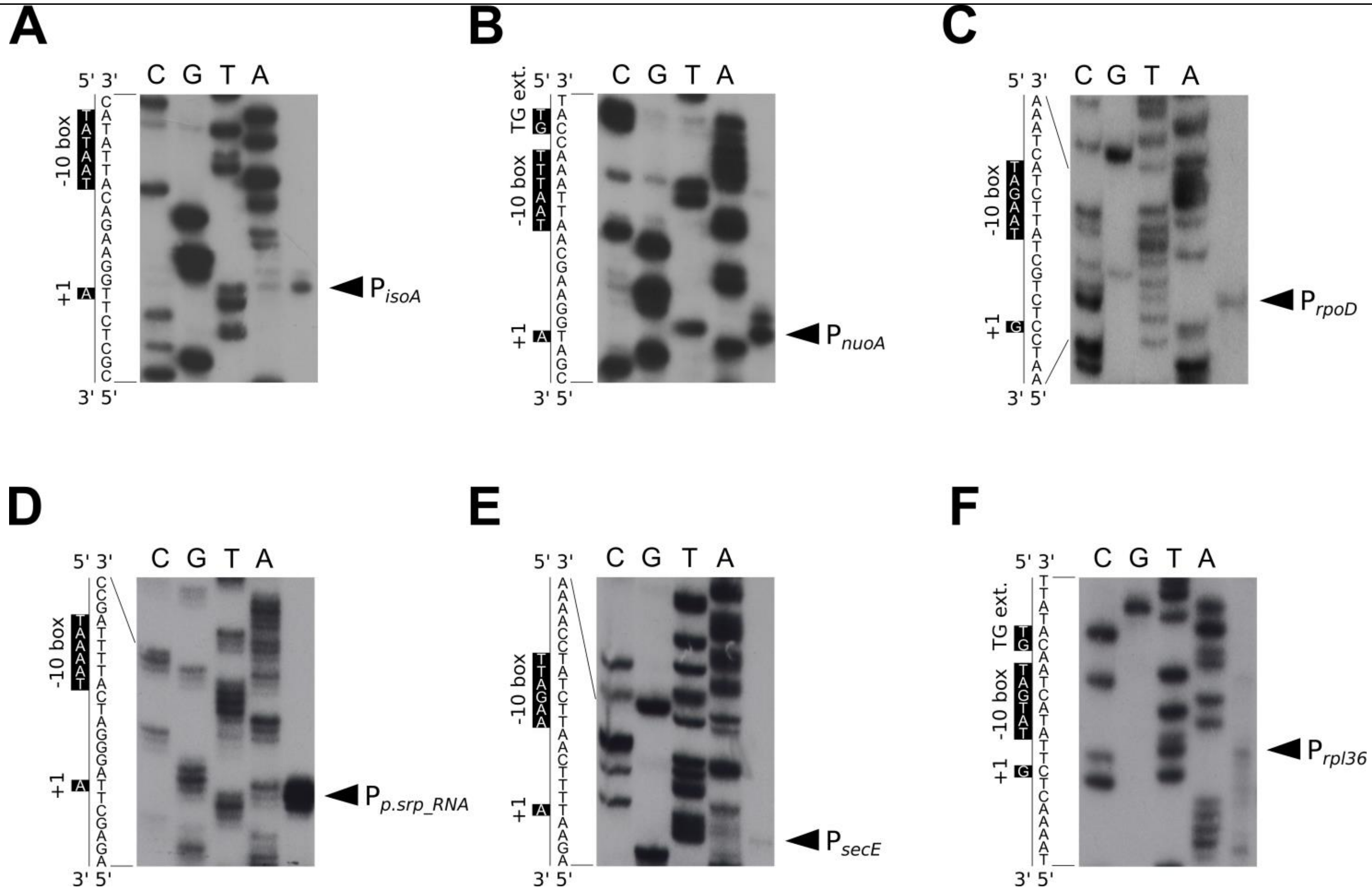

Determination of transcriptional start sites at selected promoters. Total RNA (15 µg) isolated from *H. pylori* G27 (right lanes in each panel) were hybridized to the radiolabelled oligonucleotides listed in Table S4 and elongated with AMV-reverse transcriptase. Transcriptional start sites (P_{isoA}, panel A; P_{nuoA}, panel B; P_{rpoD}, panel C; $_{Pp.srp\_RNA}$, panel D; P_{secE}, panel E, P_{rps13}, panel F) are marked with arrows on the right side of each panel. Sequence reactions (C, G, T, A) were performed with the same oligonucleotides as in the respective primer extension experiments. Nucleotide sequences (non-coding strand) with indicated promoter elements (coding strands) are reported in the left side of each panel.

Table S1

ChIP-seq	Total reads	Mapped	High quality	High quality alignment rate	Unique	PBC	NSC	RSC	Tag Quality
Input-A	6749921	6688442	6669372	98,81%	1840414	27,60%	1,02	1,33	1
Input-B	5628590	5612021	5577598	99,09%	2149154	38,53%	1,02	1,48	1
wt_HsrA_A	4253809	4224030	4211750	99,01%	1728110	41,03%	1,04	3,14	2
wt_HsrA_B	2183573	2164304	2160932	98,96%	1054816	48,81%	1,08	1,78	2

PBC=PCR Bottleneck Coefficient

NSC=Normalized Strand Cross-correlation coefficient

RSC=Relative Strand Cross-correlation coefficient

IDR consistency table	Self-consistency (THS 0.02)	Pooled sample consistency (THS 0.0025)	Consistency between replicates(THS 0.02)
**wt_HsrA_A**	63		37
**wt_HsrA_B**	59		
**Pool wt_HsrA A+B**		67	

							Gene coverage statistics		
**RNA-seq**	**Raw reads**	**Reads mapped**	**HQ reads**	**rRNA %**	**mRNA %**	**tRNA %**	**1 read covered genes %**	**Min reads covering 90% of the genes**	**Genes covered at least 50% in lenght**
**WT_HsrA_A**	3499963	3445392	3317263	4%	84%	1%	99,75%	48	96,92%
**WT_HsrA_B**	2126283	2089097	1989882	4%	83%	1%	99,50%	28	95,60%
**WT_HsrA_TET_A**	4077701	4010762	3866045	2%	87%	1%	99,50%	38	96,73%
**WT_HsrA_TET_B**	4927313	4849688	4695595	2%	87%	1%	99,43%	49	97,42%

Table S2

Extended peak annotation

	Leader gene annotation			Downstream components of the operon	
**Peak_name**	**Annotation**	**Gene_Name**	**26695_locus_tag**	**Full operon (Door predictions)**	**n. of controlled genes**
Peak_1	HPG27_RS01660	nadE	HP0329	HPG27_RS01655(lpxK)	2
	HPG27_RS01670	ilvC	HP0330	HPG27_RS01675(minD), HPG27_RS01680(minE), HPG27_RS01685(dprA), HPG27_RS01690	5
	HPG27_RS01665	tRNA-Arg	-		1
Peak_2	HPG27_RS07575	tRNA-Phe	-		1
Peak_3	HPG27_RS07130	16SrRNA	-		1
Peak_4$	HPG27_RS07980	-	-	HPG27_RS07985	2
Peak_5	HPG27_RS02200	tRNA-Pro	-		1
Peak_6	HPG27_RS05775	fldA	HP1161	HPG27_RS05770(ybeY)	2
Peak_7	HPG27_RS06075	atpE	HP1212		1
Peak_8	HPG27_RS05690	16SrRNA	-		1
Peak_9	HPG27_RS05410	galE	HP0360		1
	HPG27_RS05415	tRNA-Ile	-	HPG27_RS05420(tRNA-Ala)	2
Peak_10	HPG27_RS05865	nupC	HP1180	HPG27_RS05860(deoB)	2
	HPG27_RS05875	-	HP1181	HPG27_RS05880	2
	HPG27_RS05870	tRNA-Met	-		1
Peak_11	HPG27_RS07945	flgG2,flgG	HP1585		1
Peak_12	HPG27_RS05725	rpS16,rpsP	HP1151	HPG27_RS05710(trmD), HPG27_RS05715(rimM), HPG27_RS05720	4
Peak_13	asRNA_HPG27_RS06840	-	-		1
	HPG27_RS06835	-	-		1
Peak_14	HPG27_RS06000	secE	HP1203a	HPG27_RS05985(rpl1), HPG27_RS05990(rpl11), HPG27_RS05995(nusG)	4

Peak_15	HPG27_RS00470	fabD	HP0090	HPG27_RS00465(mtn)	2
	HPG27_RS00475	tRNA-Ser	-		1
Peak_16	HPG27_RS06065	tRNA-Leu	-		NA
	HPG27_RS06070	tRNA-Leu	-		NA
Peak_17	HPG27_RS05390	nrdB,nrdF	HP0364	HPG27_RS05395(pcm), HPG27_RS05400, HPG27_RS05405(truA)	4
	HPG27_RS05385	tRNA-Leu	-		1
Peak_18	HPG27_RS01920	tRNA-Ser	-		1
Peak_19	asRNA_HPG27_RS03935	-	-		1
Peak_20	HPG27_RS06140	hemN2,hem N	HP1226		1
	HPG27_RS06145	cytc553	HP1227		1
Peak_21	isoA	isoA	-		1
Peak_22	cncR1_Hpnc2630	cncR1	-		1
Peak_23	HPG27_RS07585	dnaA	HP1529		NA
Peak_24	HPG27_RS06525	rpl36,rpmJ	HP1297	*HPG27_RS06500(rpl17,rplQ), HPG27_RS06505(rpoA-pseudogene), HPG27_RS06510(rps4,rpsD), HPG27_RS06515(rps11), HPG27_RS06520(rps13,rpsM)	6
Peak_25	HPG27_RS00460	rpoD	HP0088	HPG27_RS00445, HPG27_RS00450(mqo), HPG27_RS00455	4
Peak_26	asRNA_HPG27_RS05530(Hpnc3560)	-	-		1
Peak_27	HPG27_RS00390	tRNA-Val	-		1
Peak_28	HPG27_RS05215	rps1,rpsA	HP0399	HPG27_RS05220, HPG27_RS05225(serA), HPG27_RS05230(ubiD), HPG27_RS05235, HPG27_RS05240, HPG27_RS05245(cheV), HPG27_RS05250(cheA), HPG27_RS05255(cheW)	9
Peak_29	putative_SRP_RNA	-	-		1
	HPG27_RS00110	-	HP0018		1
Peak_30	HPG27_RS06040	hetA	HP1206		1
Peak_31	HPG27_RS06285	bioC	HP1254		1

	HPG27_RS06290	secG	HP1255	HPG27_RS06295(frr), HPG27_RS06300(pyrE), HPG27_RS06305, HPG27_RS06310	5
Peak_32	HPG27_RS05705	rpl19,rplS	HP1147		1
Peak_33	HPG27_RS03405	flgR	HP0703		NA
Peak_34	HPG27_RS02330	hofC	HP0486		1
Peak_35	HPG27_RS00055	tRNA-Lys	-		NA
Peak_36	HPG27_RS00165	-	HP0030	HPG27_RS00160(bioD)	2
	HPG27_RS00170	uspA	HP0031	HPG27_RS00175(clpS), HPG27_RS00180(clpA), HPG27_RS00185(panD), HPG27_RS00190, HPG27_RS00195, HPG27_RS00200	7
Peak_37	HPG27_RS07170	rpl34,rpmH	HP1447		1
				Total genes of the HP1043 regulon:	92

$: located on G27 plasmid

*: inferred membership as rpmJ was absent in DOOR annotation

NA: not apply, non-promotorial peaks

Table S3

Complete list of HrsA peaks individuated by Homer on the IP replicates pool

**Peak_name**	**start:end**	**score**
Peak_1	342452:342626	33634.2
Peak_2	1570125:1570299	29290.7
Peak_3	1476498:1476672	27064.0
Peak_4$	1:126	26419.0
Peak_5	448262:448436	24526.6
Peak_6	1212453:1212627	22077.0
Peak_7	1276504:1276678	21217.1
Peak_8	1194610:1194784	20592.6
Peak_9	1140440:1140614	20533.0

Peak_10	1234366:1234540	20239.5
Peak_11	1648608:1648782	17967.3
Peak_12	1198357:1198531	17780.6
Peak_13	1411804:1411978	17187.4
Peak_14	1268342:1268516	16391.8
Peak_15	96262:96436	16366.7
Peak_16	1275920:1276094	15715.5
Peak_17	1135651:1135825	15701.4
Peak_18	395023:395197	15492.7
Peak_19	829599:829773	14406.8
Peak_20	1290665:1290839	14058.4
Peak_21	1576835:1577009	13402.5
Peak_22	536813:536987	13358.6
Peak_23	1573020:1573194	13228.3
Peak_24	1362385:1362559	12858.0
Peak_25	94349:94523	11431.6
Peak_26	1163325:1163499	11422.2
Peak_27	79140:79314	11103.6
Peak_28	1099169:1099343	10857.3
Peak_29	16420:16594	10304.9
Peak_30	1270429:1270603	10176.2
Peak_31	1318842:1319016	9864.0
Peak_32	1196544:1196718	9212.8
Peak_33	722928:723102	8986.8
Peak_34	478010:478184	8089.2
Peak_35	4434:4608	7929.2
Peak_36	31591:31765	7905.6
Peak_37	1480392:1480566	7634.1
Peak_38	1180249:1180423	7598.1
Peak_39	605388:605562	7496.1
Peak_40	3494:3668	7235.6
Peak_41	565307:565481	7125.7

Peak_42	360164:360338	7015.9
Peak_43	236202:236376	7001.8
Peak_44	1193025:1193199	6879.4
Peak_45	392093:392267	6683.2
Peak_46	1474916:1475090	6673.8
Peak_47	1085547:1085721	6341.1
Peak_48	214697:214871	6259.5
Peak_49	740257:740431	6137.1
Peak_50	414584:414758	5942.6
Peak_51	59460:59634	5865.7
Peak_52	183858:184032	5848.4
Peak_53	88918:89092	5820.2
Peak_54	1027036:1027210	5796.6
Peak_55	1552719:1552893	5732.3
Peak_56	1024984:1025158	5708.7
Peak_57	1441450:1441624	5655.4
Peak_58	201276:201450	5594.2
Peak_59	609738:609912	5550.3
Peak_60	486434:486608	5307.0
Peak_61	918864:919038	5238.0
Peak_62	128670:128844	5115.6
Peak_63	141260:141434	4917.9
Peak_64	855119:855293	4914.7
Peak_65	917889:918063	4908.5
Peak_66	588210:588384	4861.4
Peak_67	1033394:1033568	4839.4
Peak_68	1322010:1322184	4839.4
Peak_69	573065:573239	4629.1
Peak_70	971584:971758	4578.9
Peak_71	718664:718838	4455.0
Peak_72	149265:149439	4411.0
Peak_73	253325:253499	4354.5

Peak_74	966379:966553	4246.2
Peak_75	1494075:1494249	4196.0
Peak_76	1300886:1301060	4177.2
Peak_77	173406:173580	4158.4
Peak_78	1498857:1499031	3977.9
Peak_79	1465278:1465452	3863.4
Peak_80	1100311:1100485	3769.2
Peak_81	583371:583545	3745.7
Peak_82	909780:909954	3726.8
Peak_83	231004:231178	3595.0
Peak_84	1503126:1503300	3584.0
Peak_85	1460511:1460685	3571.5
Peak_86	1431410:1431584	3533.8
Peak_87	223174:223348	3527.6
Peak_88	1105721:1105895	3524.4
Peak_89	599039:599213	3515.0
Peak_90	1003793:1003967	3496.2
Peak_91	807829:808003	3488.3
Peak_92	800631:800805	3486.8
Peak_93	1155000:1155174	3453.8
Peak_94	1539595:1539769	3449.1
Peak_95	726951:727125	3389.5
Peak_96	34819:34993	3344.0
Peak_97	597916:598090	3312.6
Peak_98	658017:658191	3138.4
Peak_99	551175:551349	3097.6
Peak_100	1185982:1186156	3088.2
Peak_101	1466082:1466256	3025.4
Peak_102	898087:898261	2921.8
Peak_103	689622:689796	2797.9
Peak_104	208742:208916	2752.4
Peak_105	547113:547287	2746.1

Peak_106	67697:67871	2531.1
Peak_107	874295:874469	2145.1

$:located on G27 plasmid

Table S4

Oligonucleotides for probes generation

Name	*Sequence (5' to 3')*
0703FPF	CCCTTTCAATTGCAAAGCAAAC
0703FPR	GCCTTGCAAACTACCTTTCC
1203FPF	GCGTATCTGCTCTTTAATAGG
1203FPR	GGTTAGGTCAGTAGCTCC
1227FPF	AAATCCAGGCATAACGCTTG
1227FPR	GCGGCACAACCTTTTACAAG
sRNA17_18FPF	GATTCTAGTCATGTGAATAAAGCG
sRNA17_18FPR	GGGCTTTCTTCATAATAGAGCG
A1.4FPF	GGCAAACTACCTTAAAAGGAG
A1.4FPR	ACGCCCCAAAAGTCAAGGAC
0088FPF	CTTTAGCTTTATTTTCCTGTGTGG
0088FPR	TTACCAAACGCAAACAATTGAAAC
1296FPF	GCCCAATCCCATAAATATAGGTAAGG
1296FPR	GGATGGGAAAGTGATTGAGG
1260FPF	GCATCAGGCTCTCAAACTCC
1260FPR	CGCTCTCATTTATTACATTGACCC
0703HYF	ATATGGATCCGGGTTGAAGAGATCTTACCC
0703HYR	CCCAAGAGTATTCTAAAAGGC

1203HYF	ATATGGATCCATACATTAAAAGAATTTTCAATTCTATC
1203HYR	CCAAAACCGGTTGTTGG
1227HYF	ATATGGATCCCTCTTATTTTCACTTCATTTTTAAGC
1227HYR	GCCATTAAAGCGTTTGCG
0088HYF	ATATGGATCCGATACACCTAATCCTCTGC
0088HYR	GGTTTGTTTTTACCAAACGC
sRNA17_18HYF	ATATGGATCCGCTTAGGGATCATTTTAGCC
sRNA17_18HYR	CCGACATCAACAGAACG
A1.4HYF	ATATGGATCCGGCAAACGCTCTTGG
A1.4HYR	GAGCGCTAAAATCAGGG

Oligonucleotides for qRT-PCR analysis

Name	*Sequence (5' to 3')*	*Target CDS*
1043RTF	CAGGGTGCGGACGATTACAT	HPG27_RS02035 (HP1043 in 26695 strain)
1043RTR	AGCCCCAAAACCTCAAACGA	
0088RTF	GATAAGATACACCTAATCCTCTGCT	HPG27_RS00460 (HP0088 in 26695 strain)
0088RTR	CAAACGCAAACAATTGAAACCCT	
1203RTF	AGCCCCTAGAGAAAAATCCAAC	HPG27_RS05995 (HP1203 in 26695 strain)
1203RTR	TACGCAACGCGCTTATTTCTG	
1296RTF	TGTCAAAAGAAATGCCTACCGC	HPG27_RS06520 (HP1296 in 26695 strain)
1296RTR	ATGGCAAGGATTGCTGGTGT	
1227RTF	CGCTTGCCATGGGGTTAAGT	HPG27_RS06145 (HP1227 in 26695 strain)
1227RTR	CTTGTTGGCACCGCTTTTGA	
Feca1RTF	AGCGTGCATGGTGTCAAAAC	HPG27_RS03325 (HP0686 in 26695 strain)
FecA1RTR	AACTTCCTTGCTCCTCCAGC	
frpBRTF	TGTGAGAGGCATTGAAGACAGGCT	HPG27_RS04285 (HP0876 in 26695 strain)
frpBRTR	CGCCTTTGGTAACTTCCACGCTTT	
nixARTF	CTTACATGCTAGGGGCAAAG	HPG27_RS01865

nixARTR	CATAGGCGTTTTTGCCTTGT	(HP1077 in 26695 strain)
tRNA7RTF	TTGAAGGACAGGTGGGTGAG	tRNA7
tRNA7RTR	TCGAACCCTCGGTAACCTTG	
tRNA10RTF	CGCTCAGCCCGTAAACGAC	tRNA10
tRNA10RTR	GGCATGCAAAAAGATTACCAAAAAC	
tRNA26RTF	CAGGTGGTTAGAGCACACCC	tRNA26
tRNA26RTR	TCCCAGCTGAGCTAATTCCC	
tRNA28RTF	AATCATGGTGTCAAGAGGGGG	tRNA28
tRNA28RTR	AGCTGGTTTAGAGCGCTGG	

Oligonucleotides for primer extensions analysis

Name	*Sequence (5' to 3')*	*Target promoter*
1203FPF	GCGTATCTGCTCTTTAATAGG	P_{secE}
A1.4FPF	GGCAAACTACCTTAAAAGGAG	P_{IsoA}
1260FPF	GCATCAGGCTCTCAAACTCC	P_{nuoA}
SRP_RNA p3	CATTTTGAAGCACTCC	$P_{p.srp\_RNA}$
0088 p4	CCATAAAATAAGAACCCATGC	P_{rpoD}
1296 p2	CACATCTTTTTCACTGATGG	P_{rpl36}
